# A comparative analysis of NADPH supply strategies in *Saccharomyces cerevisiae:* Production of d-xylitol from d-xylose as a case study

**DOI:** 10.1016/j.mec.2024.e00245

**Published:** 2024-07-05

**Authors:** Priti Regmi, Melanie Knesebeck, Eckhard Boles, Dirk Weuster-Botz, Mislav Oreb

**Affiliations:** a Goethe University Frankfurt, Faculty of Biological Sciences, Institute of Molecular Biosciences, Max-von-Laue Straße 9, 60438, Frankfurt am Main, Germany; b Technical University of Munich, Chair of Biochemical Engineering, Boltzmannstr. 15, 85748, Garching, Germany

**Keywords:** NADPH supply, D-xylitol, Glucose-6-phosphate dehydrogenase, l-galactonate, *Saccharomyces cerevisiae*

## Abstract

Enhancing the supply of the redox cofactor NADPH in metabolically engineered cells is a critical target for optimizing the synthesis of many product classes, such as fatty acids or terpenoids. In *S. cerevisiae*, several successful approaches have been developed in different experimental contexts. However, their systematic comparison has not been reported. Here, we established the reduction of xylose to xylitol by an NADPH-dependent xylose reductase as a model reaction to compare the efficacy of different NADPH supply strategies in the course of a batch fermentation, in which glucose and ethanol are sequentially used as carbon sources and redox donors. We show that strains overexpressing the glucose-6-phosphate dehydrogenase Zwf1 perform best, producing up to 16.9 g L^−1^ xylitol from 20 g L^−1^ xylose in stirred tank bioreactors. The beneficial effect of increased Zwf1 activity is especially pronounced during the ethanol consumption phase. The same notion applies to the deletion of the aldehyde dehydrogenase *ALD6* gene, albeit at a quantitatively lower level. Reduced expression of the phosphoglucose isomerase Pgi1 and heterologous expression of the NADP^+^-dependent glyceraldehyde-3-phosphate dehydrogenase Gdp1 from *Kluyveromyces lactis* acted synergistically with *ZWF1* overexpression in the presence of glucose, but had a detrimental effect after the diauxic shift. Expression of the mitochondrial NADH kinase Pos5 in the cytosol likewise improved the production of xylitol only on glucose, but not in combination with enhanced Zwf1 activity. To demonstrate the generalizability of our observations, we show that the most promising strategies – *ZWF1* overexpression and deletion of *ALD6* - also improve the production of l-galactonate from d-galacturonic acid. Therefore, we expect that these findings will provide valuable guidelines for engineering not only the production of xylitol but also of diverse other pathways that require NADPH.

## Highlights

• Different NADPH supply strategies are compared in *Saccharomyces cerevisiae*.
• Example products are d-xylitol and l-galactonate.
• *ZWF1* overexpression is the most robust strategy in the diauxic batch fermentation.
• Carbon source dependencies and interferences of different strategies are explored.

## 1 Introduction

The production of most industrially relevant compound classes in metabolically engineered microbial host involves enzyme-catalyzed reductive steps. As reducing equivalents, these reactions commonly require nicotinamide adenine dinucleotide cofactors – either in the phosphorylated (NADPH) or in phosphate-free form (NADH). Although the mechanism of hydride transfer is identical for both cofactor types, their role in the cellular context is different; whereas NAD(H) is mainly used in the catabolic metabolism, most anabolic processes are dependent on NADP(H). The widely used biotechnological chassis organism *Saccharomyces cerevisiae* has specialized during evolution for fast utilization of glucose via glycolysis and subsequent alcoholic fermentation even under aerobic conditions (Piskur et al., 2006). Reduction of acetaldehyde to ethanol acts as a sink for re-oxidizing NADH, which is produced by glyceraldehyde-3-phosphate dehydrogenase (GAPDH) isoenzymes, mainly Tdh3. Overall, as a thermodynamic driving force to perpetuate fast glycolytic flux, the concentration ratio of NADH and NAD^+^ in the cytosol is kept strongly in favor of the oxidized form (Bakker et al., 2001; Canelas et al., 2008). Inversely, the NADPH/NADP^+^ couple is dominated by the reduced cofactor to facilitate the anabolic processes (Zhang et al., 2015). The latter includes biosynthetic pathways from which a wide range of biotechnologically relevant products can be derived, such fatty acids, amino acids, isoprenoids and aromatic compounds. Therefore, great research effort has been devoted to improve the supply of NADPH in metabolic engineering. The main reactions that provide NADPH in the cytosol of yeast are catalyzed by NADP^+^-dependent dehydrogenases that belong to the oxidative pentose phosphate pathway (oxPPP; glucose-6-phosphate dehydrogenase Zwf1 and 6-phosphogluconate dehydrogenase Gnd1), the cytosolic acetyl-CoA biosynthesis (acetaldehyde dehydrogenase Ald6) and anaplerotic metabolism (isocitrate dehydrogenase Idp2; relevant only on non-fermentable carbon sources) (Minard and McAlister-Henn, 2005). Therefore, the overexpression of these enzymes is an obvious target for improving NADPH supply. Among them, the overexpression of Zwf1 (the flux-controlling enzyme of the oxPPP) can be regarded as most generic, as it does not lead to production of unwanted side-products (except CO_2_). It showed positive effects in several studies, which targeted products as different as squalene (Paramasivan and Mutturi, 2017), fatty acids (Yu et al., 2018) and 1,2,4-butanetriol (Yukawa et al., 2021). In contrast, the overexpression of Ald6 leads to overproduction of acetate, which can have a negative effect on biomass production (Shiba et al., 2007; Guaragnella and Bettiga, 2021). The accumulation of acetate can be reduced if an acetate-consuming acetyl-CoA synthetase is strongly expressed together with Ald6 (Shiba et al., 2007). The feasibility of this strategy is therefore restricted to pathways that require acetyl-CoA as a building block (Kocharin et al., 2012). Similarly, overexpression of Idp2 was implemented rather in specific scenarios revolving around citrate metabolism, such as the production of α-ketoglutarate as a target molecule (Partow et al., 2017) or in a strain expressing a heterologous ATP-citrate lyase as a part of extensive metabolic engineering to increase the production of fatty acids (Yu et al., 2018).

Since the pathways involving Zwf1, Ald6 and Idp2 account for only a minor proportion of the metabolic flux in *S. cerevisiae*, it is intuitively appealing to harness glycolysis for increasing the cytosolic NADPH pool by replacing the native NAD^+^-dependent GAPDH by NADP^+^-dependent heterologous isoenzymes. Such enzymes have been described either as the non-phosphorylating (EC 1.2.1.9) type, for instance GapN from *Streptococcus mutans*, or as the phosphorylating variant (EC 1.2.1.13), represented by Gdp1 from *Kluyveromyces lactis*. Due to its reaction mechanism, the former type reduces the ATP yield of glycolysis, while the latter is energetically not different from the reactions catalyzed by the native GAPDHs in *S. cerevisiae* (Tdh1, Tdh2 and Tdh3). Both Gdp1 (Verho et al., 2003) and GapN (Bro et al., 2006) were initially used to cofactor-balance the utilization of xylose via the oxidoreductive pathway, involving an NADPH-dependent xylose reductase and an NAD^+^-dependent xylitol dehydrogenase, with a considerable success. In subsequent studies, GapN was used to improve, for instance, the production of polyhydroxybutyrate (Kocharin et al., 2013) or of fatty acids (Zhang et al., 2020) in combination with other strategies. In a strain designed to produce fatty alcohols, however, neither GapN nor Gdp1 expression exerted a positive effect on the product yields (d'Espaux et al., 2017).

All above-described approaches targeted an increase of the NADPH/NADP^+^ ratio. However, when the concentration of NAD(H) and NADP(H) is compared regardless of their oxidation state, the non-phosphorylated form is predominant by an order of magnitude (Albe et al., 1990), in accordance with the ratio of fluxes through catabolic and biosynthetic metabolism (Shen et al., 2024). The conversion of NAD(H) to NADP(H) by expressing NAD(H) kinases in the cytosol is therefore considered as an additional attractive option. For this purpose, the kinase Pos5 – naturally a mitochondrial enzyme – is regarded as particularly suitable, as it strongly prefers NADH over NAD^+^ (Outten and Culotta, 2003). It was expressed without the mitochondrial targeting sequence in several studies, with varying degree of success. A recurrent observation was a negative effect on cell growth, which is obviously a consequence of energy dissipation, since the kinase consumes ATP and decreases the amount of NADH available for respiration (Hou et al., 2009a). Whereas cytosolic Pos5 expression showed positive effects in some studies, e.g for the production of protopanaxadiol (Gao et al., 2018) and 1,2,4-butanetriol (Yukawa et al., 2021), it was described as rather detrimental by other authors (Kim et al., 2018).

**Fig. 1:**
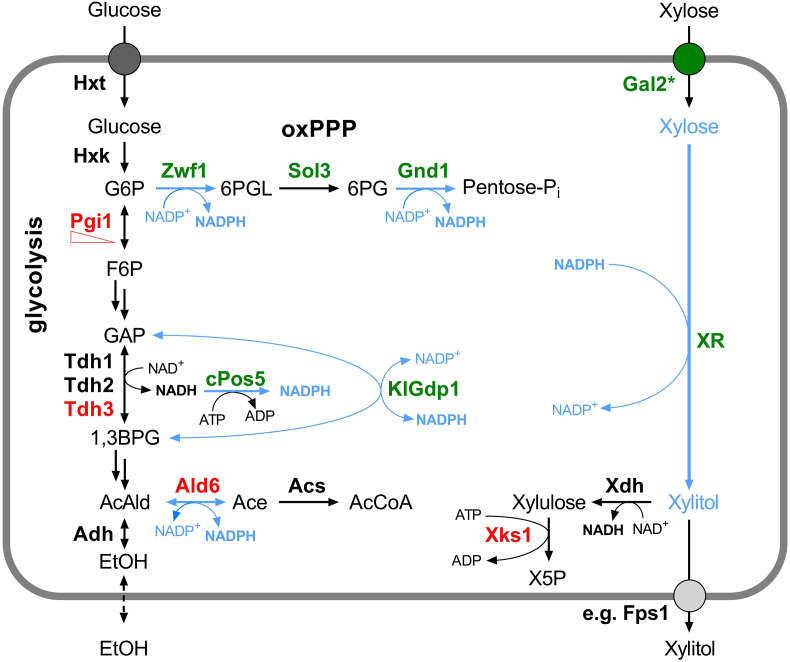
Overview of the experimental system. The reactions of the central carbon metabolism targeted in this study are shown with the involved enzymes and metabolites (G6P, glucose-6-phosphate; 6PGL, 6-phosphogluconolactone; 6 PG, 6-phosphogluconate; F6P, fructose-6-phosphate; GAP, glyceraldehyde-3-phosphate; 1,3BPG, 1,3-bisphosphoglycerate; AcAld, acetaldehyde; Ace, acetate; AcCoA, acetyl-CoA; EtOH, ethanol; X5P, xylulose-5-phosphate). Isoenzymes and transporters not relevant for this study are only abbreviated with generic names (Hxt, hexose transporters; Hxk, hexose kinase; Adh, alcohol dehydrogenase; Acs, acetyl-CoA-synthetase; Xdh, xylitol dehydrogenase). Enzymes, whose genes were deleted or downregulated (Pgi1, triangle) are shown in red, overexpressed enzymes in green. The prefix “c” (cPos5) refers to the cytosolic localization, “Kl” (KlGdp1) to the source organism of Gdp1, *K. lactis*. Blue lines depict production or consumption of NADPH through the engineered reactions. Reversible reactions are depicted with double-headed arrows, multiple reactions by double arrows. Due to the strong thermodynamic driving force of the oxPPP, its reactions are considered as practically irreversible. CO_2_ and CoA are omitted for clarity. To improve the uptake of xylose, a mutant variant of the galactose permease is overexpressed (Gal2^6SA/N376Y/M435I^, Gal2*). The NADPH-dependent reduction of xylose to xylitol is performed by xylose reductases (XR). Different transporters, such as the aquaglyceroporin Fps1 facilitate efflux of xylitol. (For interpretation of the references to color in this figure legend, the reader is referred to the Web version of this article.)

All these examples from the literature survey illustrate that many strategies to optimize NADPH supply are possible, but their comparison (not only in the quantitative sense) is difficult due to diverse experimental context. The product yields of complex heterologous pathways are usually constricted by several factors, including precursor supply, energy requirements, enzymatic bottlenecks, formation of byproducts and insufficient product secretion, all of which can limit the effects of the cofactor supply engineering. Moreover, different strain backgrounds and fermentation conditions were applied across studies. We therefore reasoned that a direct comparison of different described approaches within one study under consistent conditions would be useful to guide future engineering strategies. As a suitable readout, we have chosen the reduction of xylose to xylitol in a one-step reaction catalyzed by an NADPH-dependent xylose reductase (Hallborn et al., 1991). The uptake of xylose is possible even in native *S. cerevisiae*, but can be greatly improved by the recently described mutant Gal2^6SA/N376Y/M435I^ variant, which is, in addition to having an improved transport capacity, insensitive to competitive inhibition and degradation in the presence of glucose (Tamayo Rojas et al., 2021). These properties are important, since glucose is necessary as a co-substrate to provide NADPH for xylitol production, as well as carbon skeletons and energy for growth. The secretion of xylitol is facilitated by different transporters including the aquaglyceroporin Fps1 (Tani et al., 2016) and – like the uptake of xylose via Gal2 – energy independent. Thus, in this simple test system, the effect of NADPH supply engineering is less likely to be masked by typical constrains found in complex pathways, such as the biosynthesis of fatty acids or terpenes. An overview of the metabolic network targeted in this study is shown in Fig. 1.

## 2 Materials and methods

### 2.1 Cultivation and transformation of yeast

For the preparation of yeast competent cells, plasmid-free cells were grown in standard YPD-media (10 g L^−1^ yeast extract, 20 g L^−1^ peptone, 20 g L^−1^ glucose). Frozen competent yeast cells were prepared and yeast transformation was conducted via PEG mediated heat shock method as described previously (Gietz and Schiestl, 2007).

For auxotrophic marker selection after transformation, the cells were initially plated on solid, selective synthetic complete medium with glucose (SCD) in which uracil (-Ura) and/or leucine (-Leu) were omitted as required depending on the plasmid selection markers employed. Amino acids were added as previously described (Bruder et al., 2016). In case of dominant marker-based selection, the transformants were plated in YPD with appropriate antibiotics (100 μg mL^-1^ ClonNAT or 200 μg mL^-1^ G418) depending on the plasmid used. Yeast cultures were incubated at 30 °C. For liquid cultures, shaking was always maintained at 180 min^−1^.

### 2.2 Cultivation and transformation of Escherichia coli

Electroporation was the method used to transform shuttle plasmids into *E. coli* DH10B for DNA amplification. To culture *E. coli*, lysogeny broth (LB media) was used and the plasmid-transformed *E. coli* cells were plated on solid LB media supplemented with 100 μg mL^-1^ Ampicillin or 50 μg mL^-1^ Kanamycin, depending on the selectable marker. The temperature was maintained at 37 °C and liquid cultures were agitated at 180 min^−1^.

### 2.3 Construction of expression plasmids and CRISPR vectors

The protein sequences of Xyl1 from *Scheffersomyces (Pichia) stipitis* (Uniprot ID - P31867), XyrB from *Aspergillus niger* (NRRL3_10868) and Gre3 from *Saccharomyces cerevisiae* (Uniprot ID - P38715) were subjected to the JCat tool (http://www.jcat.de/) (Grote et al., 2005) to optimize the codon usage for expression in *S. cerevisiae.* Thereby, the codon usage was adjusted to that of highly expressed glycolytic genes (Grote et al., 2005) and the resulting codon adaptation index (CAI) value calculated by the algorithm was >0.9. The codon-optimized DNA sequences, designated as *coXYL1*, *coXYRB* and *coGRE3*, respectively, were ordered as strings from Twist Bioscience (the sequences are listed in Supplementary Table 2). The native (non-optimized) coding sequences of *GRE3* and *POS5* from *S. cerevisiae* (*ScGRE3*) and *GDP1* from *K. lactis* (*KlGDP1*) were amplified by PCR from respective genomic DNA. The non-optimized coding sequence of Gal2^6SA/N376Y/M435I^ from *S. cerevisiae* (here also referred to as GAL2*) was amplified by PCR from plasmid pRS62N_Gal2_6SA/N376Y/M435I (Tamayo Rojas et al., 2021). All expression plasmids constructed in this study except SiHV207 were based on the gap-repair procedure in yeast (Oldenburg et al., 1997). For this, the coding sequences were amplified by PCR with 30–40 basepair overhangs homologous to the *HXT7* promoter and *CYC1* terminator of the p42xH7 vector backbone. The PCR products were co-transformed with linearized vectors into CEN.PK2–1C frozen competent cells and plated on appropriate SCD selective media, depending on the auxotrophy marker used. The assembled plasmids were isolated using standard alkaline lysis protocol followed by transformation into *E. coli* for DNA amplification. Correct clones were verified by restriction digestion and sequencing. The *POS5* sequence lacking the codons of the mitochondrial targeting signal (corresponding to amino acids 1–17) was cloned into the pYTK001 entry plasmid and placed under the control of the *RNR2* promoter and *SSA1* terminator using the Golden Gate toolbox according to the established procedure (Lee et al., 2015) for the construction of SiHV207.

To construct the CRISPR vectors, guide RNA was designed using the online tool CRISPOR (http://crispor.gi.ucsc.edu/). Repair fragment (donor DNA) sequences are listed in Supplementary Table 1. If oligonucleotides were used as repair fragments, 5 μL of forward and 5 μL of reverse single-stranded DNA (each 100 μM) was mixed with 5 μL 10× annealing buffer (100 mM Tris–HCl, pH 8.0, 500 mM NaCl, and 10 mM EDTA) and 35 μL of sterile water. This mix was incubated at 95 °C for 5 min and then slowly cooled down to room temperature. The transformation was performed with 25 μL of the annealed repair fragment and 1 μg of the corresponding CRISPR-Cas9 plasmid.

All plasmids are listed in Table 1 and the primers in Supplementary Table 1.

**Table 1:** Plasmids used in this study. Under “relevant properties”, promoters and terminators are denoted by suffixes “p” and “t”, respectively.

Plasmid name	Relevant properties	References
**Expression plasmids**		
p426H7 (empty vector)	*2μ*, *URA3*, *HXT7p*^−1–392^, *CYC1t*, AmpR, pBR322-origin	Becker and Boles (2003)
p425H7 (empty vector)	*2μ*, *LEU2*, *HXT7p*^−1–392^, *CYC1t*, *AmpR*, pBR322-origin	Becker and Boles (2003)
Gal2^6SA/N376Y/M435I^-p426H7	Mutant Gal2 sequence in p426H7 vector backbone	This study
*coXYL1-p425H7*	Codon optimized *XYL1* gene from *S. stipitis* in p425H7 vector backbone	This study
*coXYRB-p425H7*	Codon optimized *XYRB* gene from *A. niger* in p425H7 vector backbone	This study
*coGRE3-p425H7*	Codon optimized *GRE3* gene from *S. cerevisiae* in p425H7 vector backbone	This study
*ScGRE3-p425H7*	Wild type sequence of *GRE3* gene from *S. cerevisiae* in p425H7 vector backbone	This study
*KlGDP1-p426H7*	Wildtype *GDP1* sequence from *K. lactis* in p426H7 vector backbone.	This study
SiHV207	[ConLS'-*RNR2p-cPOS5-SSA1t*-ConRE'-ClonNat-*LEU2*-3′Hom-kanR-ColE1-*LEU2* 5′Hom], derived from the Golden Gate system	This study
FWV169	*2μ,* AmpR*, kanMX, TEF2p-ZWF1-ZWF1t, CCW12p-SOL3-SOl3t, TEF1p-GND1-GND1t*	Wernig et al. (2021)
**CRISPR plasmids**		
pRCC-K	CRISPR vector backbone, *ROX3p-CAS9*-*CYC1t*, *SNP52p*-gRNA-*SUP4t*, *kanMX*, *2μ*, AmpR, ColE1	Generoso et al. (2016)
pRCC-N	CRISPR vector backbone, *ROX3p-CAS9*-*CYC1t*, *SNP52p*-gRNA-*SUP4t*, ClonNAT, *2μ*, AmpR, ColE1	Generoso et al. (2016)
LBGV071	CRISPR vector backbone with GFP, *ROX3p-CAS9-CYC1t, SNP52p-GFP-SUP4t, kanMX*, *2μ*, AmpR, ColE1	This study
pRCC-K_ALD6	pRCC-K-*ALD6* (CRISPR vector targeting *ALD6*)	Schadeweg and Boles (2016)
FWV157	pRCC-K-*ZWF1p* (CRISPR vector targeting *ZWF1p*)	Wernig et al. (2021)
FWV156	pRCC-K-*PGI1p* (CRISPR vector targeting *PGI1p*)	Wernig et al. (2021)
PRB4	pRCC-N-*GRE3* (CRISPR vector targeting *GRE3*)	This study
PRB53	LBGV071-*XKS1* (CRISPR vector targeting *XKS1*)	This study

### 2.4 Yeast strain construction

All strains used and constructed in this work and their relevant genotypes are listed in Table 2. PRY39, PRY48, PRY49, PRY50, PRY51, PRY52, PRY55, PRY56 and PRY63 were constructed using the CRISPR/Cas9 system (Generoso et al., 2016).

**Table 2:** Yeast strains used in this study. Under “relevant properties”, promoters and terminators are denoted by suffixes “p” and “t”, respectively; the overexpressed open reading frames are underlined. Only stable (genomic) properties are indicated.

Yeast strain name	Relevant properties	References
CEN.PK2–1C	*MATa; ura3-52; trp1-289; leu2-3112; his3Δ1; MAL2-8C; SUC2*	EUROSCARF, Frankfurt
CEN.PK2-1D	*MATα ura3-52; trp1-289; leu2-3112; his3Δ1; MAL2-8C; SUC2*	EUROSCARF, Frankfurt
CEN.PK2-1D *Δtdh3*	*MATα ura3-52; trp1-289; leu2-3112; his3Δ 1; MAL2-8C; SUC2 Δtdh3*	Linck et al. (2014)
PRY39	CEN.PK2–1C, *Δgre3*	This study
PRY48	PRY39, *ZWF1p*::*HXT7p-ZWF1*^*ΔGlu59*^(ZWF1*)	This study
PRY49	PRY48, *PGI1p::COX9p-PGI1*	This study
PRY50	PRY48, *PGI1p::RNR2p-PGI1*	This study
PRY51	PRY48, *PGI1p::REV1p-PGI1*	This study
PRY52	PRY48, *Δxks1*	This study
PRY53	PRY39, *Δleu2*::*RNR2p-cPOS5-SSA1t*, *ClonNAT*	This study
PRY54	PRY48, *Δleu2*::*RNR2p-cPOS5-SSA1t*, *ClonNAT*	This study
PRY55	PRY39, *Δald6*	This study
PRY56	PRY48, *Δald6*	This study
PRY63	PRY39, *ZWF1p*::*HXT7p-ZWF1*	This study
PRY85	PRY39, *Δura3::CCW12p-AnGATA-PGK1t-PGK1p-AnGAR1-ENO1t-kanMX*	This study
PRY86	PRY48, *Δura3::CCW12p-AnGATA-PGK1t-PGK1p-AnGAR1-ENO1t-kanMX*	This study
PRY88	PRY55, *Δura3::CCW12p-AnGATA-PGK1t-PGK1p-AnGAR1-ENO1t-kanMX*	This study

For generating yeast strains PRY53 and PRY54, the integrative vector SiHV207, derived from the Golden Gate toolbox (Lee et al., 2015), was used. SiHV207 comprises the *POS5* coding sequence lacking the N-terminal mitochondrial targeting signal (amino acids 1–17), a ClonNAT resistance cassette and flanking sequences targeting the *LEU2* locus. *POS5* is placed under the control of the comparatively weak promoter *RNR2p* (Lee et al., 2015). SiHV207 was digested with *Not*I to release the integrative cassette, which was transformed into PRY39 and PRY48, followed by plating on YPD media supplemented with ClonNAT to generate strains PRY53 and PRY54, respectively.

To delete *ALD6* in strains PRY39 and PRY48, the CRISPR vector pRCC-K_ALD6 (Schadeweg and Boles, 2016) was used.

The cassettes comprising *AnGATA* and *AnGAR1* cassettes were integrated as previously described (Harth et al., 2020) using the Golden Gate system.

All strains were verified by PCR and sequencing using appropriate primers as listed in Supplementary Table 1.

### 2.5 Fermentation in shake flasks and HPLC analysis

The plasmid-transformed yeast cells were pre-grown in a 100 mL shake flask with 30 mL of liquid SC selective media as required by the selectable marker(s) (pH 6.3 with KOH) with glucose as a carbon source. Strains were allowed to grow at 30 °C with shaking at 180 min^−1^. Cells were harvested in exponential phase, washed two times with sterile water, then transferred to 50 mL SC selective media containing 30 g L^−1^ glucose and 20 g L^−1^ xylose in a 300 mL flask. Initial OD_600_ was generally maintained at ≈0.4 and the growth was monitored by measuring OD_600_ at intervals shown in the figures. Glucose-pulsed fermentation experiments involved monitoring of the residual glucose content during the course of fermentation and supplementing glucose to 20 g L^−1^.

For HPLC analysis, 1 mL of culture sample was centrifuged at 20,000×*g* for 15 min 450 μL of the supernatant was mixed with 50 μL of 50 % (w/v) 5-sulfosalicylic acid to prevent microbial growth in the samples. The samples (10 μL) were processed in the Ultimate HPLC system (Dionex) equipped with an RI detector (Shodex) and a NucleoGel Sugar 810 FA column (Concise Separations). Separation of constituents was performed in 0.5 mM H_2_SO_4_ as liquid phase with a flow rate of 0.4 mL min^−1^ and column temperature adjusted to 30 °C. The Chromeleon 6.8 software was employed for quantifications and Prism 9 (GraphPad Software) for statistical analysis and presentation of the results.

### 2.6 Protein extraction and enzyme assays

Approximately 100 OD_600_ Units of cells from an exponentially growing culture were harvested by centrifugation (3000×*g*, 4 °C, 10 min), washed with water and stored at −80 °C until protein extraction. The cells were disrupted in Assay Buffer (50 mM Imidazole, pH 7.0, 100 mM KCl, 10 mM MgCl_2_, 0.1 mM EDTA) containing 1× concentrated Protease Inhibitor Cocktail Complete, EDTA-free (Roche Diagnostics) by shaking (10 min at 4 °C) with glass beads (0.45 mm diameter) using a Vibrax cell disruptor (Janke & Kunkel). The cell debris was removed by centrifugation (15,000×*g*, 5 min, 4 °C). Protein concentration of crude extracts was determined by the Rapid Gold BCA Protein-Assay-Kit (Pierce), using bovine serum albumin as a standard. The reaction mixtures for enzyme assays contained (final concentration) 0.67 mM NADP^+^ and 2.5 mM glucose-6-phosphate (Merck). The reactions were started by adding 20 μL of 25 mM substrate solution. The final reaction volume was 200 μL in Assay Buffer. The reduction of NADP^+^ was recorded by continuously measuring the change of the absorbance at 340 nm using an Utrospec 2100 Pro spectrometer (GE Healthcare). Specific activities were calculated as μmol substrate converted per minute (milli-Units, mU) per milligram protein.

### 2.7 Stirred tank bioreactor experiments

The preculture was produced in 1 L shake flasks without baffles with 100 mL working volume with SC-medium containing 30 g L^−1^ glucose and 20 g L^−1^ xylose. The shake flasks were inoculated with 1.5 mL of a cryostock (−80 °C) and incubated for 24 h at 30 °C and 180 min^−1^ (WiseCube, witeg Labortechnik GmbH, Wertheim, Germany). The precultured *S. cerevisiae* cells were harvested by centrifugation with 1972×*g* at 10 °C for 10 min (Rotixa 50 RS, Andreas Hettich GmbH & Co. KG, Tuttlingen, Germany). The pellet was washed two times with sterile phosphate buffer pH 6.3 (PBS) and finally suspended in 5 mL SC-media. The inoculum was drawn up in sterile syringes with a cannula and directly used for inoculation of the stirred tank bioreactors.

The batch cultivations of the *S. cerevisiae* strains were carried out in four controlled parallel stirred tank bioreactors on a 0.6 L-scale (DASGIP® Parallel Bioreactor System, Eppendorf AG, Hamburg, Germany). Each stirred tank bioreactor has a total volume of 1 L and was equipped with two Rushton turbines 3 cm and 5.5 cm above the vessel bottom. The bioreactors were filled with 600 mL SC-media. After autoclaving (121 °C, 20 min) the bioreactors were inoculated with 5 mL of the washed *S. cerevisiae* cells to achieve an initial OD_600_ of 0.4 in the bioreactors. The temperature was kept constant at 30 °C, the aeration rate (sterile air) was fixed to 0.5 vvm, and the pH was controlled with 1 M H_2_SO_4_ or 2 M KOH to pH 6.3. The initial agitation rate was set to 200 min^−1^. The agitation rate was increased automatically by the DO controller as soon as the dissolved oxygen (DO) concentration fell below 30% air saturation to prevent oxygen limitation. The CO_2_ and O_2_ concentrations in the exhaust gas were monitored (BlueVary, BlueSens Gas Sensors GmbH, Herten, Germany).

The sugar concentrations were measured by HPLC (1100 Series, Agilent Technologies Inc., Santa Clara, CA, USA) using an AMINEX® HPX87-H (300 mm × 7,8 mm) cation exchange column (Bio-Rad Laboratories GmbH, Feldkirchen, Germany) with a SecurityGuard Cartridge Carbo-H (4 × 3.0 mm) pre-column (Phenomenex Ltd., Aschaffenburg, Germany). The samples, manually withdrawn from the stirred tank bioreactors, were filtered with a 0.2 μm cellulose filter (Chromafil RC20/15 MS, Macherey-Nagel GmbH & Co., KG, Dürgen, Germany) before the HPLC analyses. 20 μL of the sterile filtered samples were injected and the separation of the sugars was carried out with a constant flow rate (0.5 mL min^−1^) of 5 mM H_2_SO_4_ as eluent. The temperature was kept constant at 65 °C. The sugars were detected with a refractive index (RI) detector (1200 Series G136A, Agilent Technologies Inc., Santa Clara, CA, USA), and quantified using respective standards.

The cell dry weight concentration (CDW) was determined gravimetrically with 1 mL sample in reaction tubes in triplicates. Each sample was centrifuged at 20,000×*g* at 10 °C for 10 min (Centrifuge 5424 R, Eppendorf SE, Hamburg, Germany) and dried at 80 °C (UN 260, Memmert GmbH, Schwabach, Germany) for at least 48 h. Afterward, the weight was measured (analytical balance XA204, Mettler-Toledo GmbH, Gieβen, Germany) and subtracted from the empty weight of the reaction tube to calculate the CDW.

## 3 Results

### 3.1 Construction of a base strain for the production of xylitol

**Fig. 2:**
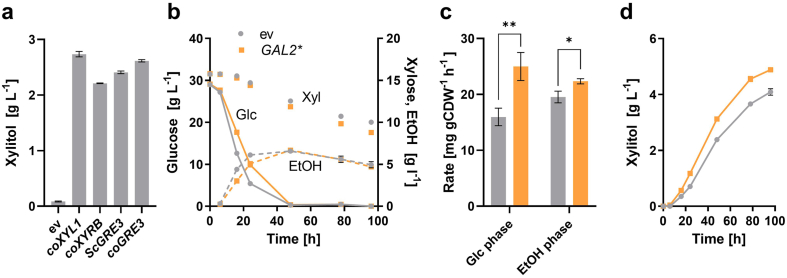
Optimization of xylose uptake and reduction. (**a**) Indicated xylose reductase genes were expressed from the multicopy plasmid p425H7 in the *Δgre3* strain PRY39. The empty vector (ev) was used as a negative control. The fermentation was performed in selective SC medium with 30 g L^−1^ glucose and 20 g L^−1^ xylose. Xylitol concentrations were determined in culture supernatants and the values measured after 48 h of fermentation are shown. In (**b-d**), the Gal2^6SA/N376Y/M435I^ variant (GAL2*) was expressed from the multicopy plasmid p426H7 in PRY39, additionally expressing *coXYL1* from p425H7. The empty vector (ev) p426H7 was used as a negative control. In (**b**), the concentrations of glucose (solid lines), xylose (symbols only) and ethanol (dashed lines) in the course of the fermentation are shown. In (**c**), the specific rates of xylose consumption were calculated for the initial 24 h (during glucose consumption, Glc phase) and in the interval from 48 h until 96 h (during ethanol consumption, EtOH phase). The rates are calculated as mg of xylose consumed in 1 h per g cell dry weight (CDW). The statistical significance was tested by a two-tailed unpaired *t*-test (*p = 0.0133; **p = 0.0061). In (**d**), xylitol titers are shown. The same color code applies for (**b-d**). All panels show mean values and standard deviations, which were determined for culture triplicates. Error bars may be smaller than the symbols. (For interpretation of the references to color in this figure legend, the reader is referred to the Web version of this article.)

We first developed a base strain that produces xylitol from xylose using glucose as a co-substrate. Based on a literature survey, the coding sequences of the following enzymes exhibiting XR activity were chosen and codon optimized: Xyl1 from *Scheffersomyces* (former *Pichia*) *stipitis* (Hallborn et al., 1991), here designated *coXYL1*; XyrB from *Aspergillus niger* (Terebieniec et al., 2021), here *coXYRB*; and Gre3 from *S. cerevisiae* (Toivari et al., 2004), here *coGRE3*. The codon-optimized sequences, as well as the native *GRE3* coding sequence from the genome of the CEN.PK strain (*ScGRE3*), which exhibits a low CAI value of 0.15, were cloned into the p425H7 plasmid backbone. The plasmids were transformed into the strain PRY39, in which the genomic *GRE3* copy (YHR104W) was deleted to minimize the background XR activity. The expression of all XR constructs led to considerably high xylitol titers; the highest values were reached with *coXYL1*, closely followed by *coGRE3* in the course of the whole fermentation, as exemplarily shown for the 48 h time point (Fig. 2a). Notably, *coGRE3* showed an increase in xylitol titers by 9% (*t*-test p-value = 0.0004) in comparison to *ScGRE3*, which demonstrates that the expression of endogenous genes can also be improved by codon optimization. Only a small amount of xylitol was produced in the empty vector control, which can be attributed to the activity of residual aldo-keto reductases, such as Ypr1 and the protein encoded by YJR096W (Träff et al., 2002).

Next, we sought to improve the uptake of xylose in the PRY39 strain background. Many of the 20 members of the hexose transporter (Hxt) family, e.g. Hxt1, Hxt5, Hxt7, Hxt11 and Gal2 are able to transport the pentose, but they exhibit a low affinity and are competitively inhibited by glucose (Leandro et al., 2009; Nijland and Driessen, 2020). We recently reported a variant of Gal2 (6SA/N376Y/M435I) that is resistant to internalization and competitive inhibition in the presence of glucose, at the same time having an improved xylose transport capacity (Tamayo Rojas et al., 2021). We therefore expressed this mutant (in the following GAL2*) from the multicopy plasmid p426H7, in addition to the *coXYL1* plasmid, and assessed its influence on the xylose consumption and xylitol production. In comparison to the empty vector control, the overexpression of GAL2* delayed the consumption of glucose (Fig. 2b) as observed in our previous work (Tamayo Rojas et al., 2021), presumably by outcompeting the glucose transporters in the plasma membrane. However, it accelerated the consumption of xylose 1.57 fold in the presence of glucose and 1.14 fold during the ethanol consumption phase (Fig. 2c). Thus, the beneficial effect is more pronounced in the presence of glucose, which is consistent with the resistance of Gal2^6SA/N376Y/M435I^ to competitive inhibition by glucose. Concomitantly, the titers of xylitol were approximately 1.22 fold higher with the GAL2* plasmid (Fig. 2d) at the terminal time point of the fermentation. Therefore, GAL2* and *coXYL1* plasmids were used in the subsequent engineering strategies.

### 3.2 Targeting the main cytosolic NADPH sources – Zwf1 and Ald6

**Fig. 3:**
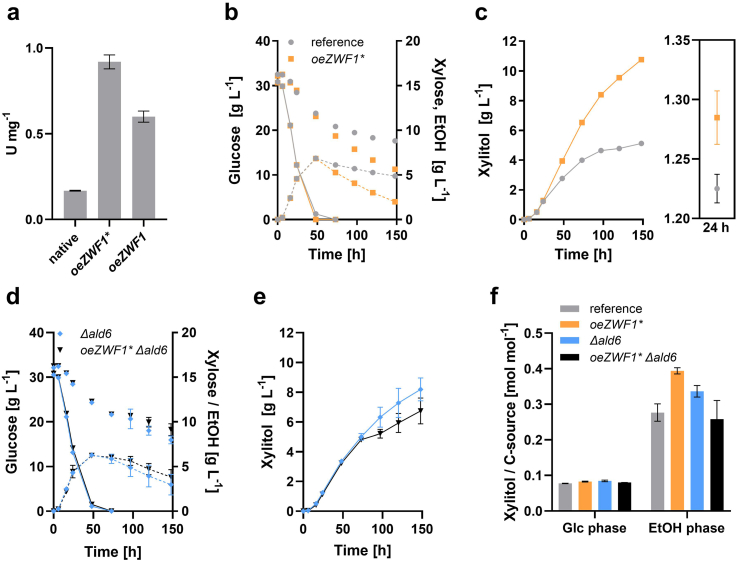
Effect of *ZWF1* overexpression and *ALD6* deletion on the production of xylitol. (**a**) G6PDH activity was measured in crude extracts from strains PRY39 (native *ZWF1* promoter, native *ZWF1* ORF), PRY48 (truncated *HXT7* promoter, ZWF1* ORF) and PRY63 (truncated *HXT7* promoter, native *ZWF1* ORF). Cells contained the GAL2* and *coXYL1* plasmids described above and were grown in selective SC medium with 20 g L^−1^ glucose for 24 h prior to protein extraction. The enzyme activities are expressed as Units per milligram of total protein. Fermentation experiments were performed with PRY39 (reference strain) and PRY48 (overexpressed ZWF1*) (**b, c**) or their *Δald6* derivatives PRY55 and PRY56 (**d, e**). All strains contained the GAL2* and *coXYL1* plasmids. The concentrations of glucose (solid lines), xylose (symbols only) and ethanol (dashed lines) in the course of the fermentation are shown in (**b**) and (**d**). Xylitol concentrations are depicted in (**c**) and (**e**), in (**c**) additionally with a zoom-in of values measured at 24 h (representative for the glucose utilization phase). In (**f**), the xylitol yields (mol per mol of consumed carbon source) were calculated in the intervals from 0 to 24 h (glucose phase) and 73–148 h (ethanol phase), respectively. The amount of *de novo* produced xylitol in each interval was divided by the correspondingly consumed amount of the respective carbon source. All panels show mean values and standard deviations, which were determined for culture triplicates. Error bars may be smaller than the symbols.

As a first approach to optimize NADPH supply, we sought to increase the flux through the oxPPP by replacing the native promoter of *ZWF1* by the truncated *HXT7p*, which is known to have a constitutive strong activity (Hauf et al., 2000). When viewing the *ZWF1* (YNL241C) SGD database entry, we realized an intriguing polymorphism; several strains, such as SK1 or RM11-1a encode a Zwf1 variant exhibiting a deletion of one glutamate residue within a di-glutamate motif (at positions 58/59) in comparison to the widely used reference strains S288C and CEN.PK. To determine if this polymorphism has some role in the activity of the enzyme, both variants were placed under the control of *HXT7p* in the genomic *ZWF1* locus of PRY39. The resulting strains PRY48 and PRY63 expressed the variant without (ZWF1*) or with Glu59 (*ZWF1*), respectively. A G6PDH assay was performed in crude extracts from the engineered strains. Compared to the parental PRY39, the strains overexpressing *ZWF1* variants showed a 5.5-fold (ZWF1*, PRY48) and 3.6-fold (*ZWF1*, PRY63) increase in activity (Fig. 3a). Thus, the deletion of Glu59 apparently led to a higher Zwf1 activity and we therefore selected PRY48 for further analyses. When fermentations were performed in glucose/xylose media, PRY48 showed, in comparison to PRY39, a slightly higher glucose consumption rate and an overall increased xylose utilization, particularly after glucose depletion (Fig. 3b). PRY48 produced the double amount of xylitol (Fig. 3c), but this difference developed mainly during the ethanol consumption phase, correlating with a concomitantly faster ethanol consumption in PRY48 (see Fig. 3b). In the presence of glucose (as shown at 24 h), there was continuously only a minor, albeit statistically significant difference (*t*-test p-value <0.02) in xylitol titers between the strains (Fig. 3c). Regarding biomass production, both strains did not differ during glucose utilization, but the ZWF1* overexpressing strain reached significantly higher OD_600_ values after the diauxic shift (Supplementary Fig. 1), paralleling the increased rate of ethanol consumption.

Considering that the major part of xylitol was produced after glucose depletion, even without ZWF1* overexpression, we asked if this might be due to the activity of the acetaldehyde dehydrogenase Ald6, the second major NADPH source in *S. cerevisiae* (Grabowska and Chelstowska, 2003). To address this question, we deleted the *ALD6* gene in the PRY39 and PRY48 background, yielding strains PRY55 and PRY56, respectively, and analyzed their performance in fermentations (Fig. 3d and e). Surprisingly, both PRY55 and PRY56 produced significantly more xylitol in comparison to the reference strain PRY39 (*t*-test p-values are 0.002 for PRY55 and 0.032 for PRY56, respectively). However, neither of them reached the titers of PRY48 (compare Fig. 3c and e). Consistently, when the molar yields of xylitol (per mol consumed carbon source) were compared, all strains reached up to four-fold higher values on ethanol compared to glucose (Fig. 3f). Collectively, these observations strongly suggest that Zwf1, rather than Ald6, is responsible for the supply of cytosolic NADPH during the ethanol consumption phase.

After having established the benefit of the increased G6PDH activity, we tested if its additional overexpression from the multicopy plasmid FWV169 (Wernig et al., 2021), together with the genes encoding the oxPPP downstream enzymes 6-phosphogluconolactonase (*SOL3*) and 6-phosphogluconate dehydrogenase (*GND1*), can further improve xylitol titers in the PRY48 strain background. However, we could not detect any positive effect in comparison to the empty vector control (Supplementary Fig. 2), suggesting that the activity of oxPPP enzymes is no longer limiting xylitol production if the genomic copy of ZWF1* is overexpressed.

### 3.3 Downregulation of the phosphoglucose isomerase gene *PGI1*

**Fig. 4:**
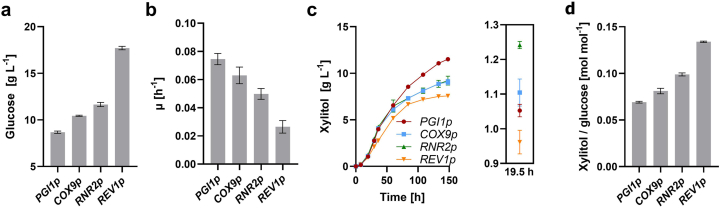
Effect of the *PGI1* downregulation. The native *PGI1* promoter (*PGI1p*) was replaced by a series of promotors exhibiting a gradual decrease in activity (*COX9p*, *RNR2p* and *REV1p*) in the PRY48 strain background. The resulting strains (all transformed with the GAL2* and *coXYL1* plasmids) were grown in selective SC medium containing 30 g L^−1^ glucose and 20 g L^−1^ xylose. Glucose and xylitol concentrations were measured in culture supernatants via HPLC analysis. In (**a**), the residual glucose concentration at 19.5 h is shown. In (**b**), the growth rates μ were calculated for the interval 8 h–19.5 h. The corresponding growth curves are shown in Supplementary Fig. 3. The time course of xylitol titers is depicted in (**c**), with a zoom-in of values measured at 19.5 h. The corresponding xylitol yields (calculated as mol of produced xylitol per mol of consumed glucose are shown in (**d**). In all panels, mean values and standard deviations were determined for culture triplicates. Error bars may be smaller than the symbols.

Since the yields of xylitol were low in the presence of glucose, we assumed that the effect of the *ZWF1* overexpression might be constrained by the competition with the strong phosphoglucose-isomerase (Pgi1) activity for the substrate G6P. To decrease the expression of the *PGI1* gene, we placed it under the control of three different promoters. One of them, *COX9p*, was reported to have an approximately nine-fold lower activity than *PGI1p* when the cells are grown on glucose (Keren et al., 2013) and was used in a previous study for improving NADPH supply (Yu et al., 2018). The other two, *RNR2p* and *REV1p*, reduce the expression level by two or three orders of magnitude, respectively, when the strong *TDH3p* is used as a reference (Lee et al., 2015). In the resulting strains (PRY49-51), the glucose consumption was indeed slowed down following the trend of the promoter strength, (Fig. 4a; the complete curves are shown in Supplementary Fig. 3). The growth rates were decreased accordingly (Fig. 4b), and the strain PRY51 (*REV1p*) showed the slowest growth (Supplementary Fig. 3). The xylitol titers (Fig. 4c) were also dependent on the used promoter; during the glucose phase (Fig. 4c), *COX9p* and *RNR2p* caused an increase in xylitol titers. With *REV1p*, there was only a low productivity, which is likely explained by the strong growth impairment in PRY51. Nevertheless, when xylitol yields (mol per mol of consumed glucose; Fig. 4d) were calculated, *REV1p* performed best and there was a clear correlation to the reduction in *PGI1* expression, as long as glucose was present in the media (as shown at 19.5 h). However, the beneficial effect was reversed after glucose depletion and the highest xylitol titers were reached with the native *PGI1p* at the terminal time point of the fermentation (Fig. 4c).

### 3.4 Expression of an NADP^+^-dependent glyceraldehyde-3-phosphate dehydrogenase

**Fig. 5:**
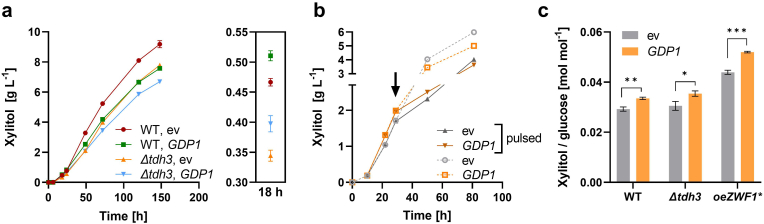
Expression of an NADP ^+^ -dependent GAPDH. (**a**) The coding sequence of *GDP1* from *K. lactis* was expressed from the multicopy plasmid p426H7 either in the wildtype CEN.PK2-1D or in the otherwise isogenic *Δtdh3* strain. The empty vector (ev) was included as a negative control. Additionally, all transformants contained the *coXYL1* plasmid. A fermentation was performed in selective SC medium containing 30 g L^−1^ glucose and 20 g L^−1^ xylose. Xylitol titers are shown, with a zoom-in of values measured at 18 h, representative for the glucose consumption phase. In (**b**) the same plasmids were introduced into PRY48 (oeZWF1*) and the cells were likewise grown in selective SC medium containing 30 g L^−1^ glucose and 20 g L^−1^ xylose. In one variant, the cells were allowed to undergo the diauxic shift; in the other (pulsed), additional glucose (20 g L^−1^) was added after 29 h (arrow), when more than 90% of the initial glucose was consumed (see Supplementary Fig. 4). The xylitol yields (mol per mol of consumed glucose) are shown in (**c**). For the WT and the *Δtdh3* strain, they were calculated in the interval 0–18 h and for PRY48 in the interval 0–22 h. The *t*-test p-values are 0.0148 (*), 0.0017 (**) and 0.0001 (***). In all panels, mean values and standard deviations were determined for culture triplicates. Error bars may be smaller than the symbols.

The above-described strategies for NADPH regeneration relied on a partial re-direction of the glucose-6-P flux towards the oxPPP. Considering that the major part of glucose still entered glycolysis, as evident from the high titers of produced ethanol (e.g. Fig. 3b), we decided to introduce a heterologous glycolytic enzyme, the glyceraldehyde-3-phosphate dehydrogenase (GAPDH) Gdp1 from *K. lactis*, into the xylitol producing strains. We first tested the effect of Gdp1 in the wildtype strain CEN.PK2-1D (without *ZWF1* overexpression) or its *Δtdh3* derivative (Linck et al., 2014). For the latter, the intention was to accumulate GAP, since Tdh3 accounts for about 60% or more of GAPDH activity in *S. cerevisiae* (McAlister and Holland, 1985a; Linck et al., 2014). In accordance with previous publications (McAlister and Holland, 1985b; Linck et al., 2014), the deletion mutant showed a growth phenotype, which was most pronounced during the glucose phase (Supplementary Fig. 4). The overall lower xylitol titers produced in the mutant can therefore be attributed to the lower cell density. The expression of *GDP1* increased xylitol titers during the early stage of fermentation in both strain backgrounds but had an opposite effect after glucose depletion (Fig. 5a). This is expectable, as GAPDH oxidizes NAD(P)H in the gluconeogenic mode. Nevertheless, we tested if Gdp1 can act synergistically with the ZWF1* overexpression; in the strain PRY48, the expression of *GDP1* further increased the production of xylitol during the glucose consumption phase (Fig. 5b). One subset of the cultures was fed with additional glucose at 29 h (when about 90% of initial glucose was consumed; see Supplementary Fig. 4) and the other was allowed to undergo the diauxic shift. The glucose-fed cells reached overall lower xylitol titers, but the beneficial effect of the *GDP1* plasmid persisted longer, thereby demonstrating the potential of this strategy under continuous glucose supply. Not only the titers, but also the yields of xylitol per consumed glucose were significantly improved in *GDP1* expressing cells, regardless of the strain background (Fig. 5c)

### 3.5 Expression of the NADH kinase Pos5 in the cytosol

**Fig. 6:**
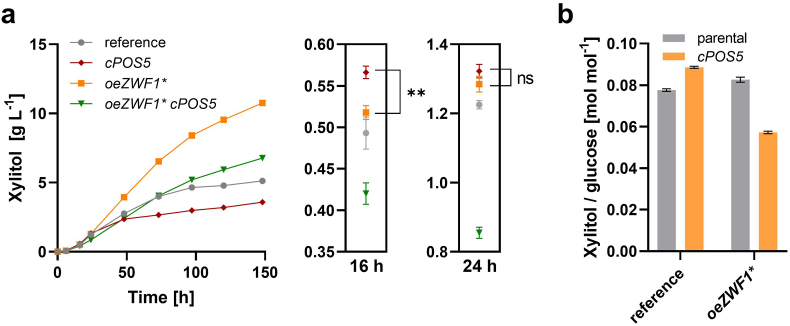
Expression of cytosolic Pos5. The NADH kinase Pos5 was expressed without the mitochondrial targeting sequence (cPos5) in the PRY39 and PRY48 strain backgrounds, yielding PRY53 and PRY54, respectively. Fermentations were performed in selective SC medium containing 30 g L^−1^ glucose and 20 g L^−1^ xylose. Xylitol titers are shown in (**a**), with a zoom-in of values measured at 16 h and 24 h (glucose consumption phase). The difference between PRY48 and PRY53 is significant at 16 h (*t*-test p-value = 0.0017, **) and non-significant (ns) at 24 h. The xylitol yields, calculated in the interval 0–24 h (mol per mol of consumed glucose), are shown in (**b**). In all panels, mean values and standard deviations were determined for culture triplicates. Error bars may be smaller than the symbols.

Whereas the strategies described above targeted the reduction of NADP^+^ to NADPH, another possibility to increase the cytosolic NADPH pool is the conversion of NADH to NADPH by an NADH kinase. The mitochondrial kinase Pos5 can phosphorylate both NADH and NAD^+^, but its preference for NADH is up to 50-fold higher compared to NAD^+^ (Outten and Culotta, 2003). For cytosolic localization, the N-terminal targeting sequence (amino acids 1–17) of Pos5 was deleted based on previous work (Strand et al., 2003). The truncated coding sequence was placed under the control of the weak *RNR2p*, since strong expression was reported to impair cell growth and/or reduce product yields (Hou et al., 2009a, 2009b; Kim et al., 2018). The expression cassette was integrated into the *leu2* locus of PRY39 and PRY48, yielding strains PRY53 and PRY54, respectively. In the PRY39 background, cytosolic Pos5 increased xylitol titers during the glucose phase and even slightly outperformed PRY48 at early time points, but this effect was lost at time points later than 24 h (Fig. 6a, Supplementary Fig. 5), similarly as shown in Fig. 4, Fig. 5. In the PRY48 background, cPos5 had a negative effect during the whole fermentation, demonstrating that it does not act synergistically with the ZWF1* overexpression (Fig. 6a). This was also reflected by the yields of xylitol per consumed glucose (Fig. 6b).

### 3.6 *ZWF1* overexpression and *ALD6* deletion improve the reduction of d-galacturonic acid

**Fig. 7:**
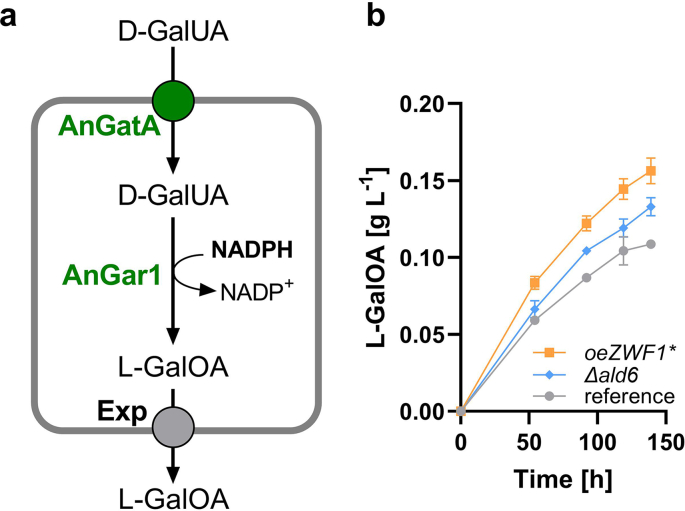
Production of l-galactonate from d-galacturonic acid. (**a**) For the uptake and reduction of d-galacturonic acid (d-GalUA), the transporter AnGatA and the NADPH-dependent reductase AnGar1 are heterologously expressed in *S. cerevisiae*. An unknown endogenous transporter of *S. cerevisiae* (Exp) exports the product l-galactonate (l-GalOA). (**b**) Expression cassettes encoding AnGar1 and AnGatA were integrated into the *ura3* locus of PRY39 (reference), PRY48 (oeZWF1*) and PRY55 (*Δald6)* strains, yielding PRY85, PRY86 and PRY88, respectively. Fermentations were performed in selective SC medium containing 30 g L^−1^ glucose and 10 g L^−1^d-galacturonic acid, buffered with 100 mM potassium phosphate, pH 6.3. l-GalOA titers are shown. Mean values and standard deviations were determined for culture triplicates. Error bars may be smaller than the symbols.

To challenge the generalizability of our findings, we decided to test another NADPH-dependent reaction in our best performing strains. We selected the reduction of d-galacturonic acid (d-GalUA) to l-galactonate (l-GalOA), which is performed by a d-GalUA reductase, such as Gar1 from *Aspergillus niger* (AnGar1). For the uptake of d-GalUA in *S. cerevisiae*, GatA, a heterologous transporter (likewise from *A. niger*; AnGatA) must be expressed. We therefore introduced expression cassettes encoding AnGar1 and AnGatA into the *ura3* locus of PRY39 (reference), PRY48 (oeZWF1*) and PRY55 (*Δald6*) strains, yielding PRY85, PRY86 and PRY88, respectively. A fermentation was performed in media containing 30 g L^−1^ glucose and 10 g L^−1^ d-GalUA, buffered with 100 mM potassium phosphate, pH 6.3, to avoid weak acid toxicity effects of d-GalUA. Although the production level of l-GalOA is significantly lower compared to that of xylitol, the strains replicated the trend shown in Fig. 3: the ZWF1* overexpressing strain produced the highest titer of xylitol, followed by the *Δald6* strain (Fig. 7). This demonstrates the generalizability of our findings in a different experimental system.

### 3.7 Biotransformation of xylose to xylitol in stirred tank bioreactors

As shown above (Fig. 3b and c), our best-performing strain, PRY48, produced a considerably high amount of xylitol, but xylose was not completely consumed even after 150 h. Moreover, at late stages of fermentation, the xylose consumption rate decreased. This cannot be explained by an inefficient xylose uptake, as we have shown previously that Gal2^6SA/N376Y/M435I^ enables a rapid and complete consumption of xylose as a carbon source even at low concentrations (Tamayo Rojas et al., 2021). In shake flask experiments, xylitol was produced mainly on ethanol, whose consumption was rather slow and incomplete (Fig. 3b). We therefore asked if the efficiency of the complete batch process could be improved in stirred tank bioreactors, which enable a high oxygen transfer rate necessary for efficient ethanol consumption. However, oxidizing conditions could promote the degradation of xylitol via xylulose, as several enzymes exhibiting NAD^+^-dependent xylitol dehydrogenase activity (e.g. Xyl2, Sor1, Sor2) are present in yeast (Toivari et al., 2004). Since deletions of all candidate genes would be laborious, we decided to delete the single xylulokinase gene *XKS1*, which should prevent the formation of xylulose-5-phosphate and thereby abolish the utilization of xylose and xylitol via the oxidoreductive route (Fig. 1). The deletion was performed in the PRY48 background, yielding strain PRY52. Both strains were transformed with GAL2* and *coXYL1* plasmids and subjected to fermentations in bioreactors.

Batch processes were performed in duplicate with the *S. cerevisiae* strains PRY48 and PRY52 in stirred tank bioreactors with SC-medium. The media contained glucose as carbon source and xylose for xylitol formation. The analysis of the carbon balances showed a carbon recovery of over 95% with both yeast strains (Supplementary Fig. 6).

**Fig. 8:**
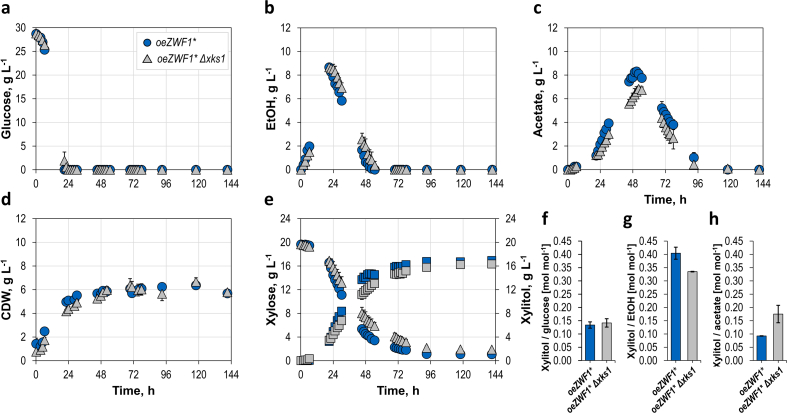
Biotransformation in stirred tank bioreactors. Batch processes were performed with PRY48 (oeZWF1*, blue) and PRY52 (oeZWF1* *Δxks1*, grey) in stirred tank bioreactors on a 0.6 L scale (duplicates). The amounts of glucose (**a**), ethanol (**b**), acetate (**c**), biomass cell dry weight (CDW) (**d**), xylose and xylitol (squares) (**e**) during replica cultivations with the respective xylitol yields (**f**–**h**) are shown. Batch fermentations were performed with SC-medium containing 30 g L^−1^ glucose, and 20 g L^−1^ xylose at pH 6.3, 30 °C, and DO > 30% air saturation. The xylitol yields with glucose were calculated in the interval 0–21 h. The xylitol yields were estimated one after the other (glucose, ethanol, acetate) from maximum concentrations to complete depletion. (For interpretation of the references to color in this figure legend, the reader is referred to the Web version of this article.)

The process performance data of both strains are summarized in Fig. 8. The glucose consumption dynamics were the same with both strains. Glucose was consumed within 21 h. Ethanol accumulation started immediately after inoculation and reached its maximum (8.7 g L^−1^ ethanol) at the time point of glucose depletion. The following phase of ethanol re-consumption was accompanied by acetate formation. After 51 h, PYR48 accumulated a maximum of 8.3 g L^−1^ acetate, whereas PRY52 produced 6.8 g L^−1^ acetate after 52 h (Fig. 8 b,c). This implies that acetate is produced from ethanol at a rate that exceeds the rate of acetyl-CoA synthesis. Acetate re-consumption started after ethanol was depleted.

The growth of both yeast strains was solely observed during glucose and ethanol consumption with maximum CDW concentrations of 5.9 g L^−1^, and 5.8 g L^−1^ after 49 h, respectively. Afterward, the CDW concentrations were constant within the estimation error.

Biotransformation of xylose was observed after a lag-phase of ∼6 h (Fig. 8e). Initially, xylose consumption and xylitol formation of both *S. cerevisiae* strains were similar. After 45 h, *S. cerevisiae* PRY48 showed an increased xylitol formation, which also matches the increased acetate formation (Fig. 8 c,e). The maximum xylitol concentrations were achieved after 93 h with final concentrations of 16.9 g L^−1^, and 16.3 g L^−1^, for strains PRY48 and PRY52 respectively (Fig. 8e). Xylitol was formed parallel to the consumption of glucose, ethanol, and acetate.

About 90% of xylose was almost stoichiometrically converted to xylitol (product yield Y_P/E_ = 0.9 mol xylitol/mol xylose) within 141 h with *S. cerevisiae* PRY48, whereas *S. cerevisiae* PRY52 showed a slightly decreased xylitol concentration (Fig. 8e) with comparable product yield (Y_P/E_ = 0.91 mol xylitol/mol xylose), possibly due to altered fluxes through the PPP. Also in the stirred tank bioreactors, a higher yield of xylitol was observed with ethanol (Y_P/Ethanol_ = 0.34–0.4 mol xylitol/mol ethanol) compared to glucose (Y_P/Glucose_ = 0.13–0.14 mol xylitol/mol glucose) as shown in Fig. 8f and g, paralleling the findings shown in Fig. 3f. Interestingly, the conversion of xylose to xylitol continued even after the full consumption of ethanol, which can be explained by the metabolization of acetate (Y_P/Acetate_ = 0.09–0.18 mol xylitol/mol acetate) (Fig. 8 h).

## 4 Discussion

The main goal of this study was a comparative analysis of different NADPH supply engineering strategies within one experimental system. The reduction of xylose to xylitol was chosen as a suitable blueprint, due to the simplicity of the process (Fig. 1) but also for the industrial relevance of the product. The results show that all tested approaches were beneficial for xylitol production, but their individual feasibility was dependent on the physiological conditions. When the whole course of the batch fermentation is considered, the overexpression of *ZWF1* enabled the highest improvement (Fig. 3c). Interestingly, a polymorphic version (ΔGlu59, here referred to as Zwf1*) exhibited a higher enzyme activity than that from the reference strains CEN.PK and S288C, which has a di-glutamate motif at positions 58/59. It is noteworthy that an allele encoding only one glutamate at the corresponding position was recently found to be associated with increased reduction of sulfate to sulfide in wine strains and the authors interpreted this as a consequence of increased NADPH supply (Guidi et al., 2024). Other authors have reported a positive correlation between Zwf1 activity and yields of xylitol, either as a target product (Kwon et al., 2006) or as an unwanted by-product of xylose fermentation to ethanol (Jeppsson et al., 2003). We observed the highest titers and yields during the ethanol consumption phase (Fig. 3c–f), which was not dependent on Ald6 activity (Fig. 3e and f). The consumption of ethanol and the concomitant production of xylitol are significantly faster in stirred-tank bioreactors, where maximum product titers of 16.9 g L^−1^ are reached (Fig. 8), probably due to improved aeration in comparison to shake flask fermentations. The pronounced effect of the ZWF1* overexpression shows that sufficient G6P is available to fuel the oxPPP even in the gluconeogenic mode of metabolism. This notion is supported by a previous report, in which an increased G6P level was observed on ethanol compared to glucose (Hou et al., 2009a). In a system-level study, it has been shown that the flux through oxPPP is downregulated after the diauxic transition in a wildtype *S. cerevisiae* strain (Zampar et al., 2013). The overexpression of ZWF1* and consumption of NADPH for xylose reduction in our approach abolished this regulatory circuit, thereby increasing the entrance of gluconeogenically derived G6P into the oxPPP even on ethanol. The 1.6-fold increased xylitol production in the *Δald6* strain compared to the PRY39 reference (compare Fig. 3c and e) was an unexpected outcome of our study, which appears counterintuitive, as Ald6 is also contributing to the cellular NADPH pool (Grabowska and Chelstowska, 2003). To test if a compensatory upregulation of *ZWF1* could be the reason for this observation, we measured the G6PDH activity in the *Δald6* strain, which indeed was higher by 16% in comparison to the reference strain (data not shown). It has been previously shown that the promoters of *ZWF1* and *ALD6* are both targeted by the transcription factor Stb5, and the cellular response to its deficiency involves differential regulation of many genes in a complex manner, dependent on the growth conditions (Ouyang et al., 2018). Thus, the upregulation of *ZWF1* may partly explain the increase in xylitol in the *Δald6* background, but the involvement of other factors cannot be ruled out. One of them may be related to the lower production of acetate, which is a known stressor to yeast cells, inducing different deleterious effects such as oxidative damage and energy depletion (Guaragnella and Bettiga, 2021). In earlier studies, the *ALD6* deletion was shown to improve the fermentation of xylose to ethanol via the oxidoreductive pathway (which involves xylitol as an intermediate), and this was attributed to a lower acetate production (Sonderegger et al., 2004; Lee et al., 2012). We show that the *ALD6* deletion also improves the reduction of d-galacturonic acid to l-galactonate, albeit with a lower efficiency than the ZWF1* overexpression (Fig. 7), which replicates the tendency seen with xylitol producing strains. Although the final clarification of the underlying mechanism is outside the scope of the present study, this suggests that the observation is generalizable and likely applicable to other NADPH consuming pathways.

All other strategies employed here showed positive effects only during the glucose consumption phase. Considering that previous studies, in which they were employed, usually did not specifically analyze the product formation in a growth stage-dependent manner, this fact may have been neglected. The downregulation of *PGI1* by promoter replacements was intended to redirect G6P towards oxPPP during glucose consumption, which apparently was successful (Fig. 4), as the xylitol yields are inversely correlated with the reported promoter strengths on glucose. The detrimental effect of promoter replacements on ethanol was somewhat unexpected, as Pgi1 was not *a priori* considered as a rate-limiting enzyme of gluconeogenesis. Nevertheless, our observation is readily explained by the reduced supply of G6P in the gluconeogenic direction and consistent with the strong effect of ZWF1* overexpression, which may create a pulling force towards oxPPP. Even though *COX9p* is known to be slightly more active (1.4-fold) on ethanol than *PGI1p*, this apparently does not compensate its (9-fold) lower transcriptional activity during glucose consumption, where biomass is produced and Pgi1 protein accumulates in the cells. We therefore propose that the downregulation of *PGI1* expression can be considered beneficial only under conditions of constant glucose supply, such as fed-batch or *simultaneous saccharification and fermentation* (SSF) regimes. In the first study known to us that combined *PGI1* downregulation with *ZWF1* overexpression (to improve the production of fatty acids), “feed beads” that release glucose slowly during fermentation and thereby prevent the formation of ethanol were used (Yu et al., 2018). Two subsequent studies employed *ZWF1* overexpression and *PGI1* downregulation in strains already expressing a heterologous phosphoketolase/phosphotransacetylase (PK/PTA) shunt to produce acetyl-CoA derivatives 3-hydroxypropionic acid (Qin et al., 2020) or octanoic acid (Wernig et al., 2021). Both reported higher product yields in the whole course of the batch fermentation (*including* ethanol consumption), in apparent contrast to observations described here. It must be considered, however, that the yields of these products are not only dependent on NADPH but also on acetyl-CoA supply via PK/PTA, whose expression likely obscured the effect of *PGI1* downregulation. For instance, downregulation of *PGI1* during the gluconeogenic mode would cause an accumulation of F6P, which is a direct substrate for PK. Furthermore, PK/PTA expression was shown to cause a major perturbation of sugar phosphate pools and a redistribution of fluxes through the central carbon metabolism (Bergman et al., 2019), which impedes a disentanglement of different synergistic or opposing effects. These examples illustrate the need for a differential examination of NADPH supply, independently of precursor supply routes, which was one of the premises of the present study.

The implementation of KlGdp1, which utilizes NAD^+^ and NADP^+^ with comparable catalytic efficiencies, showed beneficial effects and acted synergistically with ZWF1* overexpression only during the glucose consumption phase (Fig. 5). The reversal after the diauxic shift is explained by NADPH consumption by KlGdp1 in the gluconeogenic direction. Tuning the cofactor supply by expressing KlGdp1 was successfully used to improve the production of ethanol from xylose (Verho et al., 2003; Bro et al., 2006) – a scenario in which the gluconeogenic mode is not relevant. In the only remaining study known to us that reported KlGdp1 expression in *S. cerevisiae*, no beneficial effect on the production of fatty alcohols was reported (d'Espaux et al., 2017), which may be explained by other pathway limitations, as noted in the introduction.

The effects of the cytosolic Pos5 kinase expression show a differential pattern depending on the Zwf1 activity (Fig. 6). The expression of cPos5 alone increase xylitol titers even beyond the Zwf1* overexpression, but only during the glucose phase. A likely reason is a decrease in the NADH/NAD^+^ ratio by one order of magnitude after the diauxic shift (Hou et al., 2009a), which would decrease the substrate availability for Pos5. In a study designed to improve the fermentation of xylose to ethanol, the undesired accumulation of xylitol (as a pathway intermediate) was observed under anaerobic conditions (Hou et al., 2009b), which could be explained by an increased NADH accumulation. Combining Zwf1* and cPos5 expression has a detrimental effect in our study. A possible reason could be a decreased G6P accumulation when cPos5 is expressed (Hou et al., 2009a), likely as a consequence of reduced ATP yields, which would in turn reduce the substrate availability for Zwf1. Moreover, as both strategies induce a massive perturbation of cofactor pools, they may exert detrimental effects on cellular processes in a synergistic manner. Strikingly, it has been reported that Zwf1 activity is reduced upon cPos5 expression (Hou et al., 2009b). Consistent with previous studies, the growth and carbon source consumption rates of cPos5 expressing strains are slightly lower than those of their parental strains (Supplemenatary Fig. 5), suggesting that a stronger expression of cPos5 would rather negatively affect the productivity of the strains. This is likely due to the perturbations of the cofactor balance and/or to energy dissipation.

In summary, the overexpression of ZWF1* proved as a most promising strategy for a batch fermentation, followed by *ALD6* deletion. Both strategies were applicable to the reduction of xylose and d-GalUA. Increased Zwf1 activity acted synergistically with phosphoglucose isomerase downregulation and NADP^+^-dependent GAPDH - as long as glucose was present in the media - but not with a cytosolic NADH kinase. We believe that these observations shed light on the applicability of NADPH-supply optimization strategies in different scenarios and thereby have the potential to guide the engineering of diverse NADPH-dependent pathways.

## Funding

This work was supported by the German 10.13039/501100002347Federal Ministry of Education and Research [grant number 031B1048].

## CRediT authorship contribution statement

**Priti Regmi:** Writing – original draft, Methodology, Investigation, Formal analysis. **Melanie Knesebeck:** Writing – original draft, Methodology, Investigation, Formal analysis. **Eckhard Boles:** Writing – review & editing, Supervision, Resources. **Dirk Weuster-Botz:** Supervision, Funding acquisition. **Mislav Oreb:** Writing – original draft, Supervision, Project administration, Conceptualization.

## Declaration of competing interest

The authors declare that they have no known competing financial interests or personal relationships that could have appeared to influence the work reported in this paper.

## Data Availability

Data will be made available on request.
