# Supplementary material for: A comparative analysis of NADPH supply strategies in Saccharomyces cerevisiae: Production of d-xylitol from d-xylose as a case study
Source: Metab Eng Commun. 2024 Jul 5;19:e00245. doi: 10.1016/j.mec.2024.e00245 (PMC11283233; doi:10.1016/j.mec.2024.e00245)
Supplement: Multimedia component 1 [file mmc1.pdf]

Supplementary Information

A comparative analysis of NADPH supply strategies in *Saccharomyces cerevisiae:* production of D-xylitol from D-xylose as a case study

Priti Regmi[1,3], Melanie Knesebeck[2], Eckhard Boles[1], Dirk Weuster-Botz[2] and Mislav Oreb[1,*]

[1]Goethe University Frankfurt, Faculty of Biological Sciences, Institute of Molecular Biosciences, Max-von-Laue Straße 9, 60438 Frankfurt am Main, Germany

[2]Technical University of Munich, Chair of Biochemical Engineering, Boltzmannstr. 15, 85748 Garching, Germany

[3]Present Address: Tribhuvan University, Central Department of Biotechnology, TU Road 44618, Kirtipur, Kathmandu, Nepal

*corresponding author:

Dr. Mislav Oreb

Institute of Molecular Biosciences

Max-von-Laue Straße 9

60438 Frankfurt

Germany

Telephone +49 (0)69 798 29331

Telefax +49 (0)69 798 29527

E-Mail m.oreb@bio.uni-frankfurt.de

Supplementary Tables

Supplementary Table 1 | Primers used in this study

Primer	5'-3' sequence	Purpose
PRP223	AAACACAAAAACAAAAAGTTTTTTTAATTTTAATCAAAAAATGCC ATCTATCAAGTTGAAC	Construction of the *coXYL1* plasmid
PRP224	GGAGGGCGTGAATGTAAGCGTGACATAACTAATTACATGATTAAA CGAAGATTGGGATCTTG	
PRP229	AAACACAAAAACAAAAAGTTTTTTTAATTTTAATCAAAAAATGTCT AACTTGGAACACACTAAG	Construction of the *coXYRB* plasmid
PRP230	GGAGGGCGTGAATGTAAGCGTGACATAACTAATTACATGATTATTC GTCGTCACCGAAG	
PRP217	AAACACAAAAACAAAAAGTTTTTTTAATTTTAATCAAAAAATGTCT TCACTGGTTACTC	Construction of the *ScGRE3* plasmid
PRP218	GGAGGGCGTGAATGTAAGCGTGACATAACTAATTACATGATCAGG CAAAAGTGGGG	
PRP243	AAACACAAAAACAAAAAGTTTTTTTAATTTTAATCAAAAAATGTCT TCTTTGGTTACTTTGAAC	Construction of the *coGRE3* plasmid
PRP244	GGAGGGCGTGAATGTAAGCGTGACATAACTAATTACATGATTAAG CGAAAGTTGGGAACTTAC	
MOP289	CAAGAACAAACAAGCTCAAC	Sequencing primers for constructed plasmids
MOP290	ACCTAGACTTCAGGTTGTC	
PRP009	TGGTTGTAGAATCAAGCCCGGTTTTAGAGCTAGAAATAGCAAGTT AAAATAAGG	Construction of the CRIPSR plasmid PRB4
PRP010	CCGGGCTTGATTCTACAACCAGATCATTTATCTTTCACTGCGGAG	
PRP170	TTGTTCATATCGTCGTTGAGTATGGATTTTACTGGCTGGACTTTAA AAAATTTCCAATTTTCCTTTACGATTTATATTAC	Donor DNA to create PRY39
PRP171	GTAATATAAATCGTAAAGGAAAATTGGAAATTTTTTAAAGTCCAGC CAGTAAAATCCATACTCAACGACGATATGAACAA	
PRP008	CCTCGAAGTTACTACTTCTAGGG	PCR verification of strain PRY39, sequencing
PRP005	AAGCTGTTCTGTGTCTAAACAG	
PRP006	GCTTACTCCTCCTTCGGTC	
FWP328	CTCTAACCCTGGGACC	Amplification of the *HXT7p-ZWF1* cassette
PRP398	TCCTCCATGGACAATTTGGAAC	
FWP327	ACCCGTGTACATAAGCGTGAAATCACCACAAACTGTGTGTAGCTC GTAGGAACAATTTCG	Amplification of *HXT7p* for construction of PRY63
SBP176	CTCTATTCCACGAGGCATTC	
FWP328	CTCTAACCCTGGGACC	PCR verification of strains PRY48 and PRY63, sequencing
JTP240	ACAAATACACACGCAAGTAGAG	
FWP69	GAACAAACAAGCTCAACTTGTC	
SBP170	ACGTGGAATGGTGGGAAAG	
FWP322	CTGTCACCGTCAGAAAAATATGTCAATGAGGCAAGAACCGGGCT GGGCGATCTTCCTTG	Amplification of *COX9p* for

FWP323	GCCAGTTTGAAGTTAGTGAATGAGTTATTGGACATGTCTGTGTAAGTCGCTTGTAGTTAG	construction of PRY49
PRP239	TGTCACCGTCAGAAAAATATGTCAATGAGGCAAGAACCGGAGTCGAACAAGAAGCAGG	Amplification of *RNR2p* for construction of PRY50
PRP240	CAGTGGCCAGTTTGAAGTTAGTGAATGAGTTATTGGACATGGTAATTGGACAAATAAATACGTG	
PRP237	TGTCACCGTCAGAAAAATATGTCAATGAGGCAAGAACCGGGTGTTGTTATCCGATACAACCG	Amplification of *REV1p* for construction of PRY51
PRP238	CAGTGGCCAGTTTGAAGTTAGTGAATGAGTTATTGGACATCGCTGGATATGCCTAGAAATG	
MBP336	ATCGGACATGCTACCTTACG	Verification of PRY49, PRY50, PRY51
MRP151	GGTAGTTGGTCTGTTACCTGAG	
PRP255	ATGAGTCCCTCTTTATATGGGC	Sequencing primer for PRY49
PRP242	TGGATTCTTTGGTAGATAGCCAATC	Sequencing primer for PRY50
PRP241	TGAGAAAATCTGTCAACTCGTTAC	Sequencing primer for PRY51
PRP245	CTTAGAGGTCTCAGATCATCTAGAGCCTCTAACCACAGTTTTGAGACCGACGTCCTG	Construction of the CRISPR plasmid PRB53
PRP246	CAGGACGTCGGTCTCAAAACTGTGGTTAGAGGCTCTAGATGATCTGAGACCTCTAAG	
PRP247	CTCTCGAGAAAAACAAAAGGAGGATGAGATTAGTACTTTAAATATGTTTGAATAATTTATCATGCCCTGACAAGTACACA	Donor DNA to create PRY52
PRP248	TGTGTACTTGTCAGGGCATGATAAATTATTCAAACATATTTAAAGTACTAATCTCATCCTCCTTTTGTTTTTCTCGAGAG	
PRP187	ATCCTGCTGTAGTTATGGC	PCR verification of PRY52
PRP188	TTCTGTTACCTTGTTAACCGAATC	
Hdp556	AGAGGTTTCCAACACAATG	
Afp38	TTTCGTAAGCTTCTGGTTC	
JWP092	TATTTTGTGTATATGACGGAAAGAAATGCAGGTTGGTACATGTATTCTGATAGTATGTGTTTGTGTATGTTAAAGATGTT	Donor DNA to create PRY55 and PRY56
WGP535	AACATCTTTAACATACACAAACACATACTATCAGAATACATGTACCAACCTGCATTTCTTTCCGTCATATACACAAAATA	
TWRP34	GTCAAGCCTGGCGTGTTTAACAAG	PCR verification of PRY55 and PRY56
TWRP35	AAGAAGGCACAAGCCTGTTCTC	
SiHP060	GGGCGGATTACTACCGTT	PCR verification of strains PRY85, PRY86 and PRY88
SiHP061	GTAATGTTATCCATGTGGGC	
SiHP062	AGAGCACTTGAATCCACTGC	
SiHP063	GATTTGGTTAGATTAGATATGGTTTC	

Supplementary Table 2 | Synthetic genes used in this study

Gene	ORF Sequence (5'-3')	Reference
coXYL1	ATGCCATCTATCAAGTTGAACTCTGGTTACGACATGCCAGCTGTTGGTTTCGGTTGTTGG<br>AAGGTTGACGTTGACACTTGTTCTGAACAAATCTACAGAGCTATCAAGACTGGTTACAGA<br>TTGTTCGACGGTGCTGAAGACTACGCTAACGAAAAGTTGGTTGGTGCTGGTGTTAAGAAG<br>GCTATCGACGAAGGTATCGTTAAGAGAGAAGACTTGTTCTTGACTTCTAAGTTGTGGAAC<br>AACTACCACCACCCAGACAACGTTGAAAAGGCTTTGAACAGAACTTTGTCTGACTTGCAA<br>GTTGACTACGTTGACTTGTTCTTGATCCACTTCCCAGTTACTTTCAAGTTCGTTCCATTG<br>GAAGAAAAGTACCCACCAGGTTTCTACTGTGGTAAGGGTGACAACTTCGACTACGAAGAC<br>GTTCCAATCTTGGAAACTTGGAAGGCTTTGGAAAAGTTGGTTAAGGCTGGTAAGATCAGA<br>TCTATCGGTGTTTCTAACTTCCCAGGTGCTTTGTTGTTGGACTTGTTGAGAGGTGCTACT<br>ATCAAGCCATCTGTTTTGCAAGTTGAACACCACCCATACTTGCAACAACCAAGATTGATC<br>GAATTCGCTCAATCTAGAGGTATCGCTGTTACTGCTTACTCTTCTTTCGGTCCACAATCT<br>TTCGTTGAATTGAACCAAGGTAGAGCTTTGAACACTTCTCCATTGTTCGAAAACGAAACT<br>ATCAAGGCTATCGCTGCTAAGCACGGTAAGTCTCCAGCTCAAGTTTTGTTGAGATGGTCT<br>TCTCAAAGAGGTATCGCTATCATCCCAAAGTCTAACACTGTTCCAAGATTGTTGGAAAAC<br>AAGGACGTTAACTCTTTCGACTTGGACGAACAAGACTTCGCTGACATCGCTAAGTTGGAC<br>ATCAACTTGAGATTCAACGACCCATGGGACTGGGACAAGATCCCAATCTTCGTTTAA	This study
coXYRB	ATGTCTAACTTGGAACACACTAAGAAGGTTTACACTTTGAACACTGGTGACAAGATCCCA<br>GCTGTTGGTTTGGGTACTTGGCAATCTAAGCCAAACGAAGTTAGAGAAGCTGTTAAGAAC<br>GCTTTGTTGAAGGGTTACAGACACATCGACACTGCTTTGGCTTACGGTAACGAAGCTGAA<br>GTTGGTCAAGGTATCAAGGACTCTGGTGTTCCAAGAGAAGAAATCTGGGTTACTACTAAG<br>TTGGACAACCCATGGCACCACAGAGTTGCTGAAGGTATCGACTCTTCTTTGAAGGACTTG<br>GGTTTGGACTACGTTGACTTGTACTTGGTTCACTGGCCATCTTCTACTGACCCAAACGAC<br>TTGAAGAAGCACTTGCCAGACTGGGACTTCATCAAGACTTGGCAAGAAATGCAAAAGTTG<br>CCAGCTACTGGTAAGGTTAGAAACATCGGTGTTTCTAACTTCGGTATCAAGAACTTGGAA<br>AAGTTGTTGAACGACCCATCTTGTAAGATCGTTCCAGCTGTTAACCAAATCGAATTGCAC<br>CCAAACAACCCATCTCCAAAGTTGGTTGCTTACAACACTTCTAAGGGTATCCACTCTACT<br>GGTTACTCTTGTTTGGGTTCTACTAACTCTCCATTGTACAAGGACGAAACTTTGTTGAAG<br>TTGGCTGAAAAGAAGGGTAAGACTCCACAACAAGTTTTGTTGTTGTGGGGTGTTCAAAAG<br>GGTTGGTCTGTTATCCCAAAGTCTGTTTCTAAGTCTAGAATCGACGCTAACTTCGAATTG<br>GACGGTTGGGAATTGACTGCTGAAGAAATCGAACAATTGGACAACTTGAAGGACAGATTC<br>AAGGTTTGTGGTGACGACTGGTTGCCAGTTAAGGTTTTCTTCGGTGACGACGAATAA	This study
coGRE3	ATGTCTTCTTTGGTTACTTTGAACAACGGTTTGAAGATGCCATTGGTTGGTTTGGGTTGT<br>TGGAAGATCGACAAGAAGGTTTGTGCTAACCAAATCTACGAAGCTATCAAGTTGGGTTAC<br>AGATTGTTCGACGGTGCTTGTGACTACGGTAACGAAAAGGAAGTTGGTGAAGGTATCAGA<br>AAGGCTATCTCTGAAGGTTTGGTTTCTAGAAAGGACATCTTCGTTGTTTCTAAGTTGTGG<br>AACAACTTCCACCACCCAGACCACGTTAAGTTGGCTTTGAAGAAGACTTTGTCTGACATG<br>GGTTTGGACTACTTGGACTTGTACTACATCCACTTCCCAATCGCTTTCAAGTACGTTCCA<br>TTCGAAGAAAAGTACCCACCAGGTTTCTACACTGGTGCTGACGACGAAAAGAAGGGTCAC<br>ATCACTGAAGCTCACGTTCCAATCATCGACACTTACAGAGCTTTGGAAGAATGTGTTGAC<br>GAAGGTTTGATCAAGTCTATCGGTGTTTCTAACTTCCAAGGTTCTTTGATCCAAGACTTG<br>TTGAGAGGTTGTAGAATCAAGCCAGTTGCTTTGCAAATCGAACACCACCCATACTTGACT<br>CAAGAACACTTGGTTGAATTCTGTAAGTTGCACGACATCCAAGTTGTTGCTTACTCTTCT<br>TTCGGTCCACAATCTTTCATCGAAATGGACTTGCAATTGGCTAAGACTACTCCAACTTTG<br>TTCGAAAACGACGTTATCAAGAAGGTTTCTCAAAACCACCCAGGTTCTACTACTTCTCAA<br>GTTTTGTTGAGATGGGCTACTCAAAGAGGTATCGCTGTTATCCCAAAGTCTTCTAAGAAG<br>GAAAGATTGTTGGGTAACTTGGAAATCGAAAAGAAGTTCACTTTGACTGAACAAGAATTG<br>AAGGACATCTCTGCTTTGAACGCTAACATCAGATTCAACGACCCATGGACTTGGTTGGAC<br>GGTAAGTTCCCAACTTTCGCTTAA	This study

Supplementary Figures

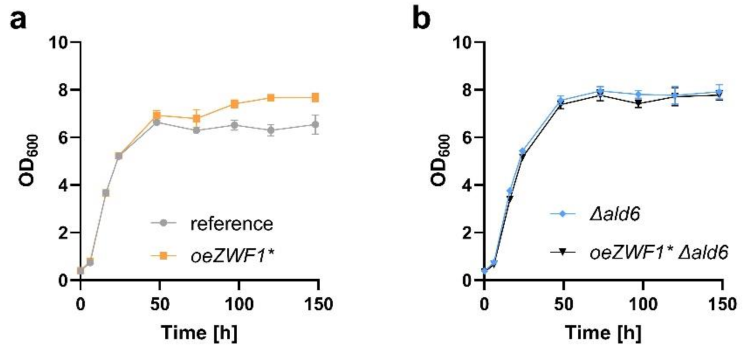

Supplementary Figure 1. Effect of *ZWF1* overexpression and *ALD6* deletion on the growth. Fermentation experiments were performed with PRY39 (reference strain) and PRY48 (overexpressed *ZWF1**) (**a**) or their *Δald6* derivatives PRY55 and PRY56 (**b**). The relevant modifications are indicated in the legend. All strains contained the *GAL2** and *coXYL1* plasmids. The optical density of the culture (OD_{600}) was recorded in the course of the fermentation. Both panels show mean values and standard deviations, which were determined for culture triplicates. Error bars may be smaller than the symbols.

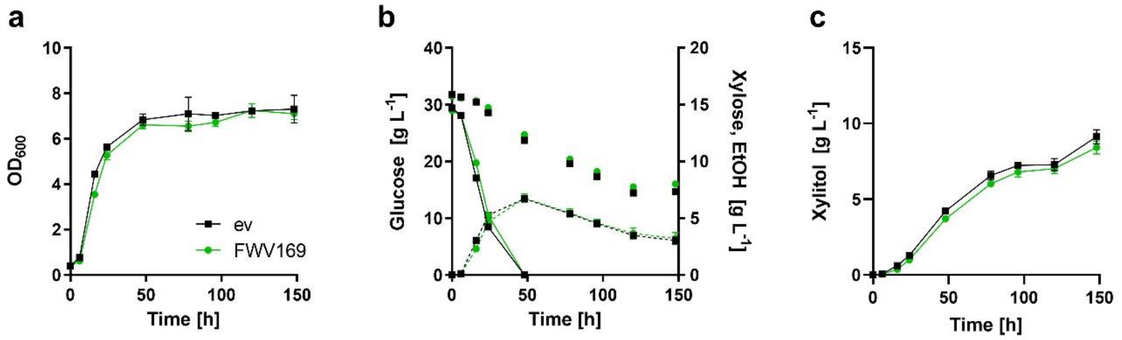

Supplementary Figure 2. Plasmid-based expression of the oxidative pentose phosphate pathway genes. The plasmid FWV169 with expression cassettes encoding *ZWF1*, *SOL3* and *GND1* genes was introduced into the strain PRY48. The cells transformed with the empty vector (ev) were used as a negative control. Additionally, the cells contained the *GAL2** and *coXYL1* plasmids. Fermentations were performed in selective SC medium containing 30 g L^{-1} glucose and 20 g L^{-1} xylose. The optical density (OD_{600}) of the cultures is shown in (**a**). The concentrations of glucose (solid lines), xylose (symbols only) and ethanol (dashed lines) in the course of the fermentation are shown in (**b**). Xylitol concentrations are depicted in (**c**). All panels show mean values and standard deviations, which were determined for culture triplicates. Error bars may be smaller than the symbols.

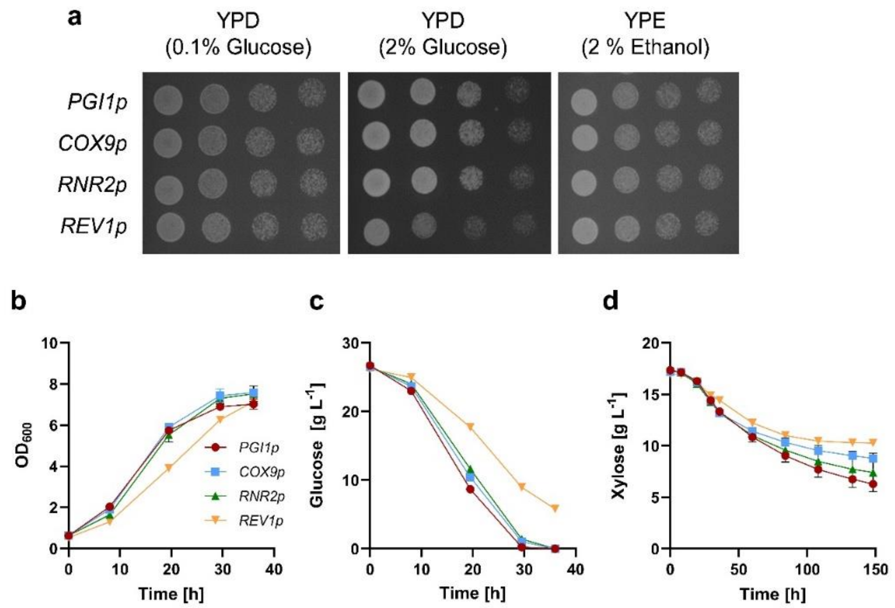

Supplementary Figure 3. Effect of the *PGI1* downregulation. The native *PGI1* promoter (*PGI1p*) was replaced by a series of promotors exhibiting a gradual decrease in activity (*COX9p*, *RNR2p* and *REV1p*) in the PRY48 strain background. (**a**) A dilution series (10^0-10^{-3}) of the resulting strain was spotted onto the agar plates with YP medium supplemented with indicated carbon sources. The plates were incubated for two days at 30°C. In (**b-d**), the strains were transformed with the *GAL2** and *coXYL1* plasmids and grown in selective SC medium containing 30 g L^{-1} glucose and 20 g L^{-1} xylose. The optical density (**b**), glucose (**c**) and xylose (**d**) concentrations were measured in the course of the fermentation. The growth curves are shown only until 36 h for a clearer visualization of the differences during the glucose consumption phase. In all panels, mean values and standard deviations were determined for culture triplicates. Error bars may be smaller than the symbols.

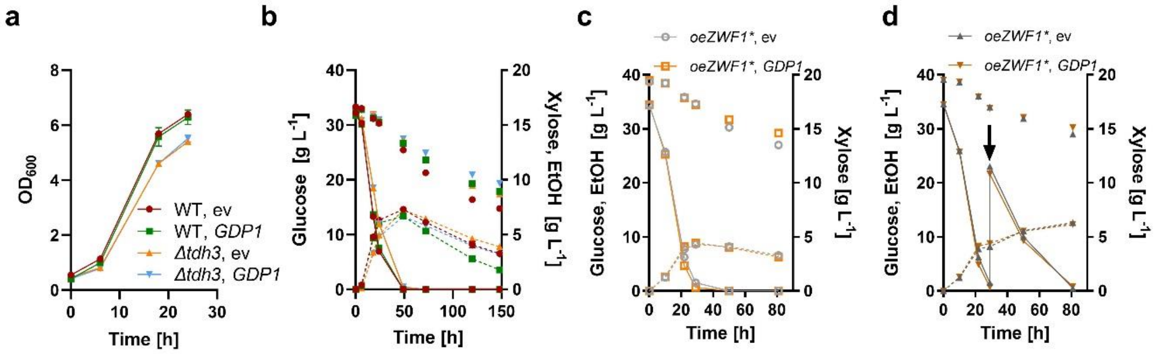

Supplementary Figure 4. Introduction of a NADP$^+$-dependent GAPDH. The coding sequence of *GDP1* from *K. lactis* was expressed from the multicopy plasmid p426H7 either in the wildtype CEN.PK2-1D (WT), in the otherwise isogenic *Δtdh3* strain or in PRY48 (*oeZWF1**). In all strains, the empty vector (ev) was included as a negative control. Additionally, all transformants contained the *coXYL1* plasmid. A fermentation was performed in selective SC medium containing 30 g L^{-1} glucose and 20 g L^{-1} xylose. For the WT and the *Δtdh3* strain, growth curves (**a**) are shown to demonstrate the growth phenotype caused by the deletion of *TDH3*. In (**b**)-(**d**), the concentrations of glucose (lines), xylose (symbols only) and ethanol (dashed lines) are shown for the indicated strains. In (**c**), a batch fermentation with PRY48 was performed, and in (**d**) an additional glucose pulse was applied at the 29 h time point (arrow). Note that the xylitol titers corresponding to (**c**) and (**d**) are shown together in Figure 5B for direct comparison.

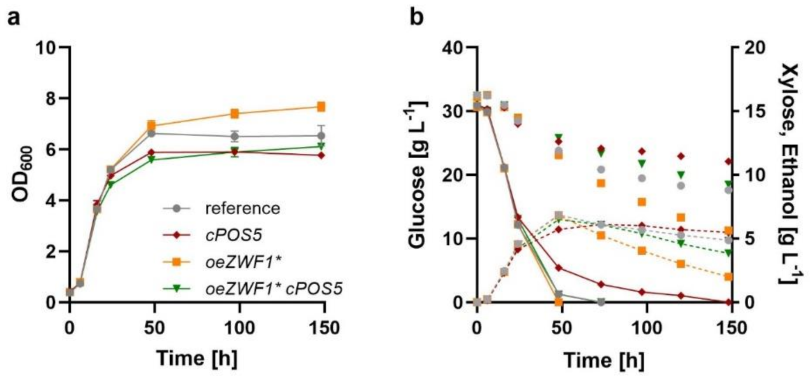

Supplementary Figure 5. Expression of cytosolic Pos5. The NADH kinase Pos5 was expressed without the mitochondrial targeting sequence in the PRY39 (reference) and PRY48 (*oeZWF1**) strain backgrounds, yielding PRY53 and PRY54, respectively. The relevant modifications are indicated in the legend. Fermentations were performed in selective SC medium containing 30 g L^{-1} glucose and 20 g L^{-1} xylose. Growth curves (OD_{600}) are shown in (**a**). In (**b**), the concentrations of glucose (lines), xylose (symbols only) and ethanol (dashed lines) are shown. The mean values and standard deviations were determined for culture triplicates. Error bars may be smaller than the symbols.

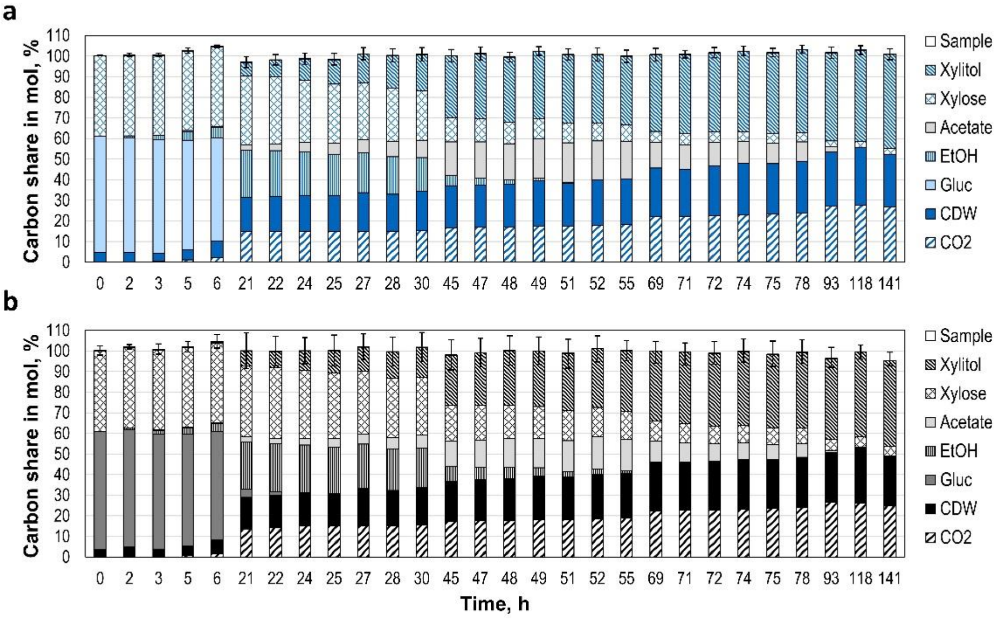

Supplementary Figure 6. Carbon balance of processes with the PRY48 (**a**) and PRY52 strain (**b**) in duplicates each with their minima and maxima values as error bars. Fermentations were performed in SC-medium containing 30 g L^{-1} glucose and 20 g L^{-1} xylose, the pH was controlled at pH 6.3, the temperature constant at 30 °C, and the dissolved oxygen concentration regulated above 30% air saturation by increasing the agitation rate. The bars show the shares of each component from the total carbon content in the process. The carbon shares are shown for CO_2, cell dry weight (CDW), glucose (Gluc), ethanol (EtOH), acetate, xylose, xylitol, and the sampling (sample) were also taken into account.
